# Long-Term Monitoring of the Antibody Response to a SARS-CoV-2 Infection

**DOI:** 10.3390/diagnostics11101915

**Published:** 2021-10-16

**Authors:** Václav Šimánek, Ladislav Pecen, Hana Řezáčková, Ondřej Topolčan, Karel Fajfrlík, Dalibor Sedláček, Robin Šín, Monika Bludovská, Petr Pazdiora, David Slouka, Radek Kučera

**Affiliations:** 1 Department of Immunochemistry Diagnostics, University Hospital in Pilsen, 309 55 Pilsen, Czech Republic; simanek@fnplzen.cz (V.Š.); rezackovah@fnplzen.cz (H.Ř.); topolcan@fnplzen.cz (O.T.); kucerar@fnplzen.cz (R.K.); 2 Department of Microbiology, University Hospital in Pilsen, 309 55 Pilsen, Czech Republic; fajfrlik@fnplzen.cz; 3 Department of Infectious Diseases and Travel Medicine, University Hospital in Pilsen, 309 55 Pilsen, Czech Republic; sedlacek@fnplzen.cz (D.S.); sinr@fnplzen.cz (R.Š.); 4 Faculty of Medicine in Pilsen, Institute of Pharmacology and Toxicology, Charles University, 323 00 Pilsen, Czech Republic; Monika.Bludovska@lfp.cuni.cz; 5 Faculty of Medicine in Pilsen, Institute of Epidemiology, Charles University, 301 00 Pilsen, Czech Republic; pazdiora@fnplzen.cz; 6 Department of Otorhinolaryngology, University Hospital in Pilsen, Faculty of Medicine in Pilsen, Charles University, 309 55 Pilsen, Czech Republic; slouka@fnplzen.cz

**Keywords:** antibody, SARS-CoV-2, COVID-19, time period, nucleocapsid protein, serological diagnostics, immunoassay

## Abstract

A group of 110 patients from the West Bohemian region who had been infected with COVID-19 was monitored for the purposes of this study. We focused on cases of mild or moderate COVID-19; statistically the most likely to occur. Day zero was defined as the day on which a positive PCR test was first established. The mean length of observation was 6.5 months, the maximum length 12 months. The first blood samples were taken from a smaller cohort during the 1–3 months following the first positive PCR test. We assumed that SARS-CoV-2 antibodies would be present during this period and therefore a limited number of samples were taken for the purpose of detecting antibodies. More samples were collected, starting 4 months after the first positive PCR test. A subsequent set of blood samples were drawn, mostly 6 months after the first ones. Our study confirmed the presence of total IgG SARS-CoV-2 antibodies up to 1 year after the onset of the disease. The peak of antibody production was observed in the third month after the first positive PCR test. A mathematical estimate of the median duration of antibody positivity was calculated to be 18 months from the onset of the COVID-19 infection.

## 1. Introduction

Understanding the antibody response, including the long-term presence of severe acute respiratory syndrome coronavirus 2 (SARS-CoV-2) antibodies in the human body is essential for developing strategies to fight against COVID-19 [1]. The clinical picture of COVID-19 can vary widely: from an asymptomatic course of disease to mild or severe symptoms [2]. Detection of antibodies in human serum can help us confirm whether a patient’s immune system has encountered SARS-CoV-2 virus and whether they have developed an immune response against it. A patient who has developed antibodies is more likely to be protected from future infection [3,4]. Clinical data suggest that the course of disease in patients who were reinfected with other types of coronavirus was weaker and sometimes they remained immune to the disease 6–12 months after the first infection [4,5].

The aim of this study was to monitor the presence of the SARS-CoV-2 antibodies in patients with the most frequent course of the COVID-19 disease (mild or moderate) and to define a curve depicting the production of SARS-CoV-2 antibodies over time.

## 2. Material and Methods

### 2.1. Group of Patients

The selected patients were monitored during the period of March 2020–April 2021. The group consisted of 110 patients (51 men, 59 women). All patients enrolled in the study were examined in the University Hospital in Pilsen and came from the West Bohemian region. They had mild or moderate symptoms; COVID-19 disease was confirmed using PCR. Day zero of the observation was defined as the day on which a positive PCR test was first established. The clinical characteristics of the patient group are shown in Table 1.

Informed consent was obtained from all the patients enrolled in the study. The study was approved by the Ethics Committee of University Hospital and Medical Faculty in Pilsen on 8th August 2020 (approval number 354/2020).

### 2.2. Serum Samples

Peripheral venous blood was collected using a VACUETTE blood collection system (Greiner Bio-one Company, Kremsmünster, Austria). Serum was separated by centrifugation at 1300× *g*, aliquoted into two aliquots of 500 µL each and frozen at −80 °C. One aliquot was used for SARS-CoV-2 antibody determination, a second aliquot was used as back-up. The aliquots were thawed only once, just prior to the analyses.

The first blood samples used to determine anti-SARS-CoV-2 antibodies were taken from a smaller cohort of patients 1–3 months after the first positive PCR test (16 patients, see Table 2). During this period the presence of anti-SARS-CoV-2 antibodies was presumed and therefore only a limited number of samples were taken to ensure that the production of antibodies is taking place. More samples were collected starting in month 4 following the positive PCR test. The second set of blood samples were drawn mostly 6 months after the first set. In a smaller group of patients, a third set of samples was taken approximately one year after COVID-19 disease had been confirmed. In total, 242 blood samples were drawn. The overview of the sample collection is shown in Table 2.

### 2.3. Immunoassays

An automated chemiluminescence (CLIA) assay ELECSYS Anti-SARS-CoV-2 was used for the detection of the total immunoglobulin (total Ig: IgM, IgG, and IgA) antibodies against the nucleocapsid protein (NP) (F. Hoffmann-La Roche, Basel, Switzerland). The Roche assay reports the results as cut-off index (COI), without units. Assay characteristics are shown in Table 3.

## 3. Statistical Methods

SAS, V. 9.4 (SAS Institute Inc., Cary, NC, USA) was used for all statistical analysis. Summary statistics for discrete variables are expressed as frequency counts and percentages, for continuous variables as means and standard deviations or medians and quartiles, where appropriate. For some variables, such as age range, the minimum and maximum are also presented. Visualizations such as mean-course plots (means ± standard deviations) and spaghetti plots are presented. Due to a relatively low number of patients, we advocated for the use of data visualization methods. Different sampling times was statistically solved by calculating of the time interval from the first positive PCR test to sampling date in days, then this time interval was converted to months and rounded to whole numbers. For the lowest months 1st, 2nd, 3rd, it was tested that the data are not biased in one direction from an integer multiple of the month. Gender and age dependencies of the measured parameters were also analyzed. The first-order elimination rate constant of the levels of SARS-CoV-2 antibodies was used. It leads to the fitting of the linear regression model for each patient where the dependent variable is the natural logarithm of the level of SARS-CoV-2 antibodies and the independent variable is the time after the peak at 3 months. By estimating the slope and intercept in this model, we estimate the duration of antibody positivity for each patient with 95% confidence interval (CI). The half-life of the level of antibodies is the time required to reach a 50% reduction in concentration.

## 4. Results

**Figure 1:**
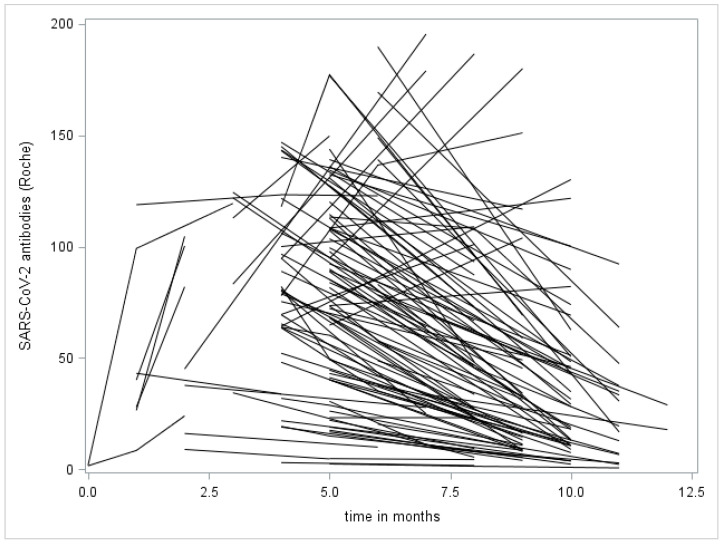
Individual curves of the levels of SARS-CoV-2 antibodies.

The individual levels of SARS-CoV-2 antibodies are shown in Figure 1. Point “zero” is the day of the first positive PCR test. The “x” axis represents the time from the first positive PCR test in months, the “y” axis represents the serum concentration of the SARS-CoV-2 antibodies. The mean length of observation, as well as the mean length of antibody response was 6.5 months; the longest observation period, as well as the longest detected presence of antibodies, was 12 months. After an initial increase in production, antibodies decreased in most individuals during the observed period. This decrease was gradual, and even 1 year after infection, antibody levels remained positive. Only one patient in the monitored group exhibited a sharp decrease in antibody values during the period of observation (below 1.0 COI).

**Figure 2:**
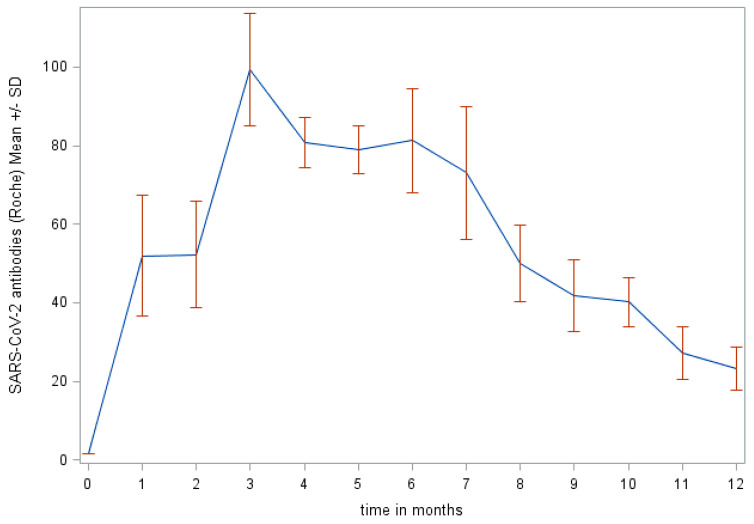
SARS-CoV-2 antibodies in the observed group of patients over the course of a year.

The detailed description of SARS-CoV-2 antibody levels in individual months is shown in Table 4; graphic display in Figure 2. The peak of the antibody production was reached in the monitored group of patients during the third month following the first positive PCR test.

Subsequently, we tried to estimate the duration of antibody positivity. The half-life of antibody concentration was 84 days (95% CI = 65–108). The rate of the decrease was calculated using the natural logarithm and then converted to the decadic logarithm. The rate of the decrease was 8.0 × 10^−3^, (95% CI = 6.1 × 10^−3^ − 9.0 × 10^−3^) per day. This means that in our data, the logarithm is reduced by about 1 over a period of 4.11 months, (95% CI = 3.56–4.62) (i.e., from 100 to 10 or from 10 to 1). Value 10 was chosen as the minimum antibody level required for immunity which we used to calculate the individual rate of antibody decrease because in our scale this value is sufficiently deep in the positive zone. Thus, from the first PCR test to level 10 (COI), immunity lasts a median of 18.0 months, (95% CI = 14.8–22.3). However, this is under the conditions of the expected standard exponential decline (which corresponds to first-order kinetics). In fact, the decline seems to be slightly faster and it appears that level 10 (COI) will be exceeded earlier; i.e., the median immunity will be between 15 and 16 months.

We did not observe a statistically significant difference in the production of antibodies between men and women. We did not observe a correlation between the production of antibodies and age.

The results divided by age categories and genders, as well as the detailed data of the group of patients, are available in the Supplementation Material (Suppl. Table S1: anti-SARS-CoV-2 Total Ig Roche—Summary statistics by age category; Suppl. Figure S1: anti-SARS-CoV-2 Total Ig Roche—Mean course plot by age category; Suppl. Table S2: anti-SARS-CoV-2 Total Ig Roche—summary statistics by gender; Suppl. Figure S2: Anti-SARS-CoV-2 Total Ig Roche—summary statistics by gender; Suppl. Table S3: Individual patient data.). Suppl. Table S1 shows the division into age groups with the goal of differentiating between children + adolescents, adults and elderly populations.

## 5. Discussion

The COVID-19 pandemic has profoundly changed the healthcare system. Initial triage of patients upon entrance to the medical facility, antigen and PCR testing, alongside with health status questionnaires, have become everyday routine practice [6].

An understanding of the mechanism and duration of the antibody response during and after a COVID-19 infection is essential in the development of a successful strategy for the management of pandemic. As COVID-19 continues to spread worldwide, our understanding of the antibody response is increasing, as is evidenced by a systematic review of 150 studies describing the antibody response to SARS-CoV-2 [7].

Literature findings in antibody dynamics confirm the typical immunological paradigm: the start of antibody production, increase, peak, plateau, decrease and persistence at lower levels [8]. In our study, an automated CLIA assay of the total antibody measurement was used. This method differs from the pure IgG assay: a higher sensitivity of total antibody assay is visible at the beginning of the antibody production, making the earlier increase in the antibody levels observable thanks to IgM detection.

This is advantageous when observing patients long-term, as it enables us to compare the kinetics of antibodies found using the total antibody assay with the kinetics of antibodies found using IgG assays. IgM disappears from the blood within a few weeks.

A number of studies confirm that IgM become undetectable within 5 weeks from disease onset [9,10]. We focused mainly on the production of antibodies from the fourth month onwards, which meant that the kinetics of the antibodies were no longer affected by IgM.

According to a number of studies, IgG peaks in the seventh- or eighth-week following disease onset and then, after a certain period during which their production plateaus, the levels start to decline; the speed of the decline varies from individual to individual [10,11,12,13,14]. The kinetics of the antibody production observed by us corresponded with the literature. It is clearly visible in Figure 2 that after an initial increase, the peak of the antibody production was reached at the third month following the first positive PCR test and, after that, the production gradually decreased in most individuals. This trend is also shown in Figure 1.

The length of observation in our study corresponded with the length of the confirmed presence of antibodies in the monitored patients. The mean length of observation, as well as the mean length of antibody response, was 6.5 months; the longest observation period, as well as the longest detected antibody response, was 12 months. The half-life of antibodies concentration was 84 days (95% CI = 65–108). This is comparable with another literature findings [15].

The duration of the antibody response is hard to compare with the literature because the longest antibody response is limited by the length of the follow-up period among published studies. As the body of research on SARS-CoV-2 increases, we are also seeing that the confirmed length of the antibody response increases [16,17].

Due to the fact that our follow-up periods are limited at the moment, we used a mathematical log-linear regression model to calculate the rate of the decrease in antibodies and to estimate of period during which the antibodies will remain within the positive ranges. The rate of the decrease was calculated as 8.0 × 10^−3^, 95% CI (6.1 × 10^−3^, 9.0 × 10^−3^) per day. It was difficult to decide which level of antibodies is appropriate as the cut-off point for the half-life calculation because we observed large differences in the rate of decline between individuals. Taking into account the individual rates of decrease in antibodies, we chose value 10 (COI), because in our scale this value is sufficiently deep in the positive zone.

We took the rate of the decrease in antibody concentration into account in a subset of patient who had at least three antibody assessments after the 3rd month following the first positive PCR test and we extrapolated that level 10 (COI) will be reached by a median (50%) of the patients between 15 and 16 months after the COVID-19 infection onset. In order to confirm this model, we need more patient with three or more antibody assessments. Under the conditions of the expected standard exponential decline (which corresponds to the first-order kinetics) immunity lasts a median of 18 months, 95% CI (14.8, 22.3).

Figure 1 shows more than the decreases in the individual antibody concentrations: It also shows an increase in antibody levels during 1–3 months after the first positive PCR test. This follows the model of antibody kinetics described above.

In our group of patients, a minority of patients exhibited a gradual increase in antibody concentration after month 4 or 5. This trend was observed in 12 patients (11%). This increase in antibody concentrations is probably caused by repeated contact with the virus, as is described in the literature [18]. We tried to clarify the observed increase in antibody levels directly with the individual patients. All 12 patients were interviewed and only one patient confirmed that they had suffered a repeated infection with clinical symptoms. The remaining 11 patients were probably repeatedly infected as well, which led to a rise in antibody production despite the patients remaining asymptomatic.

The correlation between age and antibody production remains disputed. Some studies have reported a positive correlation between antibody level and age [19,20], while other studies found no correlation [21,22]. Aware of the extreme challenges faced by the senior population during the pandemic [23], we focused on a separate evaluation of the antibody response and antibody production dynamic in the group of over 60-year-olds and compared our observations with the younger age categories of the population. In our previous research [24], the positive correlation between age and the production of neutralization antibodies was described. This finding was at the border of statistical significance (*p* = 0.040). In our current study, the positive correlation between age and antibody production was not significant if data are tested by timepoint (month) from the first positive test. When we focused on the peak of antibody production, we observed a slightly lower level of production at the peak of antibody production in young patients (20 and under), as well as a lower level at the peak of production in old patients (60 and older). Larger population studies are necessary to confirm this fact.

Despite information to the contrary in the literature [25,26], we failed to confirm a difference in antibody production between genders.

## 6. Conclusions

Our study showed that IgG SARS-CoV-2 antibodies can remain present in the body up to one year after the onset of the disease.

The peak of the antibody production was observed during the third month following the first positive PCR test. The mean length of antibody response was 6.5 months; the longest detected antibody response was 12 months. The half-life of antibody concentration was 84 days. When we take the rate of the decrease in antibody concentration into account, we can extrapolate that level 10 (COI) will be reached by a median of patients (50%) under the conditions of the expected standard exponential decline (which corresponds to the first-order kinetics) at 18.0 months, 95% CI (14.8, 22.3).

## Figures and Tables

**Table 1:** Clinical characteristics of the patient group.

Characteristic	Category	N (%)
Gender	Female	59 (53.6)
	Male	51 (46.4)
	Total	110
Age	Median [range]	49 (4–73)
Clinical symptoms	fever ≥37.5 °C	56 (50.9)
	dry cough	32 (29.1)
	difficulty breathing or shortness of breath	28 (25.5)
	loss of taste or smell	47 (42.7)
	headache	54 (49.1)
	tiredness	78 (70.9)
Risk factors	obesity	16 (14.5)
	smoking (last 10 years)	15 (13.6)
	diabetes	9 (8.2)
	cardiovascular disease	10 (9.1)
Flu vaccination	for season 2019	7 (6.4)

**Table 2:** Overview of the sample collection.

Month After PCR Test	1st Blood Draw	2nd Blood Draw	3rd Blood Draw	Total
0	2	0	0	2
1	5	2	0	7
2	4	3	1	8
3	5	0	1	6
4	34	2	0	36
5	48	7	0	55
6	10	7	1	18
7	2	10	0	12
8	0	22	1	23
9	0	16	10	26
10	0	29	3	32
11	0	10	5	15
12	0	2	0	2
Total	110	110	22	242

**Table 3:** Assay characteristics of CLIA ELECSYS Anti-SARS-CoV-2.

Manufacturer	Methodology	Antigen Used	Cut-Off Index (COI)		Manufacturer’s Catalog Number
			Negative	Positive	
Roche	CLIA	Total Ig NP	<1.0	≥1.0	09 203 095 190

**Table 4:** Levels of SARS-CoV-2 antibodies in the monitored group of patients over the course of the observation period.

Month After PCR Test	Count of Samples	Mean	Standard Deviation	Minimum	Lower Quartile	Median	Upper Quartile	Maximum
0	2	1.6	0.1	1.5	1.5	1.6	1.7	1.7
1	7	52.0	15.5	8.4	26.4	40.1	99.2	118.8
2	8	52.3	13.5	8.8	19.9	41.3	91.1	104.6
3	6	99.3	14.4	34.3	83.1	116.1	121.9	124.4
4	36	80.7	6.4	2.9	63.6	79.4	103.1	146.9
5	55	79.0	6.2	2.3	40.2	83.8	112.8	177.2
6	18	81.3	13.2	9.9	37.6	63.6	136.6	189.8
7	12	73.2	16.9	10.9	30.6	64.6	84.8	195.5
8	23	50.0	9.7	1.3	9.3	33.6	87.2	186.6
9	26	41.7	9.2	3.9	9.1	22.2	51.4	180.0
10	32	40.2	6.2	2.2	11.2	30.3	56.8	130.1
11	15	27.1	6.7	0.6	6.5	19.3	37.4	92.1
12	2	23.3	5.5	17.8	17.8	23.3	28.8	28.8

## Data Availability

Data are available by the corresponding author.

## Supplementary Materials

The following are available online at https://www.mdpi.com/article/10.3390/diagnostics11101915/s1, Figure S1: Anti-SARS-CoV-2 Total Ig Roche—summary statistics by age category, Figure S2: Anti-SARS-CoV-2 Total Ig Roche—summary statistics by gender, Table S1: anti-SARS-CoV-2 Total Ig Roche—summary statistics divided into age category, Table S2: anti-SARS-CoV-2 Total Ig Roche—summary statistics by gender, Table S3: Individual patient data.

## References

[1] Gozalbo-Rovira R., Gimenez E., Latorre V., Francés-Gómez C., Albert E., Buesa J., Marina A., Blasco M.L., Signes-Costa J., Rodríguez-Díaz J. (2020). SARS-CoV-2 Antibodies, Serum Inflammatory Biomarkers and Clinical Severity of Hospitalized COVID-19 Patients. J. Clin. Virol..

[2] Gaebler C., Wang Z., Lorenzi J.C.C., Muecksch F., Finkin S., Tokuyama M., Ladinsky M., Cho A., Jankovic M., Schaefer D. (2021). Evolution of Antibody Immunity to SARS-CoV-2. Nature.

[3] Sekine T., Perez-Potti A., Rivera-Ballesteros O., Strålin K., Gorin J.-B., Olsson A., Llewellyn-Lacey S., Kamal H., Bogdanovic G., Muschiol S. (2020). Robust T Cell Immunity in Convalescent Individuals with Asymptomatic or Mild COVID-19. Cell.

[4] Kellam P., Barclay W. (2020). The Dynamics of Humoral Immune Responses Following SARS-CoV-2 Infection and the Potential for Reinfection. J. Gen. Virol..

[5] Lega S., Naviglio S., Volpi S., Tommasini A. (2020). Recent Insight into SARS-CoV2 Immunopathology and Rationale for Potential Treatment and Preventive Strategies in COVID-19. Vaccines.

[6] Tolone S., Gambardella C., Brusciano L., del Genio G., Lucido F.S., Docimo L. (2020). Telephonic Triage before Surgical Ward Admission and Telemedicine during COVID-19 Outbreak in Italy. Effective and Easy Procedures to Reduce In-Hospital Positivity. Int. J. Surg..

[7] Post N., Eddy D., Huntley C., van Schalkwyk M.C.I., Shrotri M., Leeman D., Rigby S., Williams S.V., Bermingham W.H., Kellam P. (2020). Antibody Response to SARS-CoV-2 Infection in Humans: A Systematic Review. PLoS ONE.

[8] Chaudhury S., Hutter J., Bolton J.S., Hakre S., Mose E., Wooten A., O’Connell W., Hudak J., Krebs S.J., Darden J.M. (2021). Serological Profiles of Pan-Coronavirus-Specific Responses in COVID-19 Patients Using a Multiplexed Electro-Chemiluminescence-Based Testing Platform. PLoS ONE.

[9] Xiao A.T., Gao C., Zhang S. (2020). Profile of Specific Antibodies to SARS-CoV-2: The First Report. J. Infect..

[10] Li K., Huang B., Wu M., Zhong A., Li L., Cai Y., Wang Z., Wu L., Zhu M., Li J. (2020). Dynamic Changes in Anti-SARS-CoV-2 Antibodies during SARS-CoV-2 Infection and Recovery from COVID-19. Nat. Commun..

[11] Isho B., Abe K.T., Zuo M., Jamal A.J., Rathod B., Wang J.H., Li Z., Chao G., Rojas O.L., Bang Y.M. (2020). Persistence of Serum and Saliva Antibody Responses to SARS-CoV-2 Spike Antigens in COVID-19 Patients. Sci. Immunol..

[12] Sun B., Feng Y., Mo X., Zheng P., Wang Q., Li P., Peng P., Liu X., Chen Z., Huang H. (2020). Kinetics of SARS-CoV-2 Specific IgM and IgG Responses in COVID-19 Patients. Emerg. Microbes Infect..

[13] Adams E., Ainsworth M., Anand R., Andersson M.I., Auckland K., Baillie J.K., Barnes E., Beer S., Bell J.I., Berry T. (2020). Evaluation of Antibody Testing for SARS-CoV-2 Using ELISA and Lateral Flow Immunoassays. MedRxiv.

[14] Huang J., Mao T., Li S., Wu L., Xu X., Li H., Xu C., Su F., Dai J., Shi J. (2020). Long Period Dynamics of Viral Load and Antibodies for SARS-CoV-2 Infection: An. Observational Cohort Study. MedRxiv.

[15] Khoury D.S., Cromer D., Reynaldi A., Schlub T.E., Wheatley A.K., Juno J.A., Subbarao K., Kent S.J., Triccas J.A., Davenport M.P. (2021). Neutralizing Antibody Levels Are Highly Predictive of Immune Protection from Symptomatic SARS-CoV-2 Infection. Nat. Med..

[16] Carsetti R., Zaffina S., Piano Mortari E., Terreri S., Corrente F., Capponi C., Palomba P., Mirabella M., Cascioli S., Palange P. (2020). Different Innate and Adaptive Immune Responses to SARS-CoV-2 Infection of Asymptomatic, Mild, and Severe Cases. Front. Immunol..

[17] Marcos-Jiménez A., Sánchez-Alonso S., Alcaraz-Serna A., Esparcia L., López-Sanz C., Sampedro-Núñez M., Mateu-Albero T., Sánchez-Cerrillo I., Martínez-Fleta P., Gabrie L. (2021). Deregulated Cellular Circuits Driving Immunoglobulins and Complement Consumption Associate with the Severity of COVID-19 Patients. Eur. J. Immunol..

[18] Dembrovszky F., Váncsa S., Farkas N., Erőss B., Szakó L., Teutsch B., Bunduc S., Nagy R., Dohos D., Kiss S. (2021). Immunoglobulin Response and Prognostic Factors in Repeated SARS-CoV-2 Positive Patients: A Systematic Review and Meta-Analysis. Viruses.

[19] Ma H., Zeng W., He H., Zhao D., Jiang D., Zhou P., Cheng L., Li Y., Ma X., Jin T. (2020). Serum IgA, IgM, and IgG Responses in COVID-19. Cell Mol. Immunol..

[20] Madariaga M.L.L., Guthmiller J.J., Schrantz S., Jansen M.O., Christensen C., Kumar M., Prochaska M., Wool G., Durkin-Celauro A., Oh W.H. (2021). Clinical Predictors of Donor Antibody Titre and Correlation with Recipient Antibody Response in a COVID-19 Convalescent Plasma Clinical Trial. J. Intern. Med..

[21] Luchsinger L.L., Ransegnola B., Jin D., Muecksch F., Weisblum Y., Bao W., George P.J., Rodriguez M., Tricoche N., Schmidt F. (2020). Serological Analysis of New York City COVID19 Convalescent Plasma Donors. medRxiv.

[22] Brochot E., Demey B., Touzé A., Belouzard S., Dubuisson J., Schmit J.-L., Duverlie G., Francois C., Castelain S., Helle F. (2020). Anti-Spike, Anti-Nucleocapsid and Neutralizing Antibodies in SARS-CoV-2 Inpatients and Asymptomatic Individuals. Front. Microbiol..

[23] Gambardella C., Pagliuca R., Pomilla G., Gambardella A. (2020). COVID-19 Risk Contagion: Organization and Procedures in a South Italy Geriatric Oncology Ward. J. Geriatr. Oncol..

[24] Šimánek V., Pecen L., Krátká Z., Fürst T., Řezáčková H., Topolčan O., Fajfrlík K., Sedláček D., Šín R., Pazdiora P. (2021). Five Commercial Immunoassays for SARS-CoV-2 Antibody Determination and Their Comparison and Correlation with the Virus Neutralization Test. Diagnostics.

[25] Zeng F., Dai C., Cai P., Wang J., Xu L., Li J., Hu G., Wang Z., Zheng F., Wang L. (2020). A Comparison Study of SARS-CoV-2 IgG Antibody between Male and Female COVID-19 Patients: A Possible Reason Underlying Different Outcome between Sex. J. Med. Virol..

[26] Fafi-Kremer S., Bruel T., Madec Y., Grant R., Tondeur L., Grzelak L., Staropoli I., Anna F., Souque P., Fernandes-Pellerin S. (2020). Serologic Responses to SARS-CoV-2 Infection among Hospital Staff with Mild Disease in Eastern France. EBioMedicine.

