# Supplementary material for: Long-Term Monitoring of the Antibody Response to a SARS-CoV-2 Infection
Source: Diagnostics (Basel). 2021 Oct 16;11(10):1915. doi: 10.3390/diagnostics11101915 (PMC8534661; doi:10.3390/diagnostics11101915)
Supplement: Supplementary file 1 [file diagnostics-11-01915-s001.zip › diagnostics-1364312-supplementary.pdf]

Supplementary Materials: Long-Term Monitoring of the Antibody Response to a SARS-CoV-2 infection

Václav Šimánek, Ladislav Pecen, Hana Řezáčková, Ondřej Topolčan, Karel Fajfrlík, Dalibor Sedláček, Robin Šín, Monika Bludovská, Petr Pazdiora, David Slouka and Radek Kučera

S1. Results

Suppl. Figure S1 is a visualization of the mean-course plot of antibody levels by age. Antibody levels follow a different course in different age groups. However, these differences were not statistically significant *(note: if variability is not shown in the figure below that is an indication that only one measurement was taken per group/point in time).*

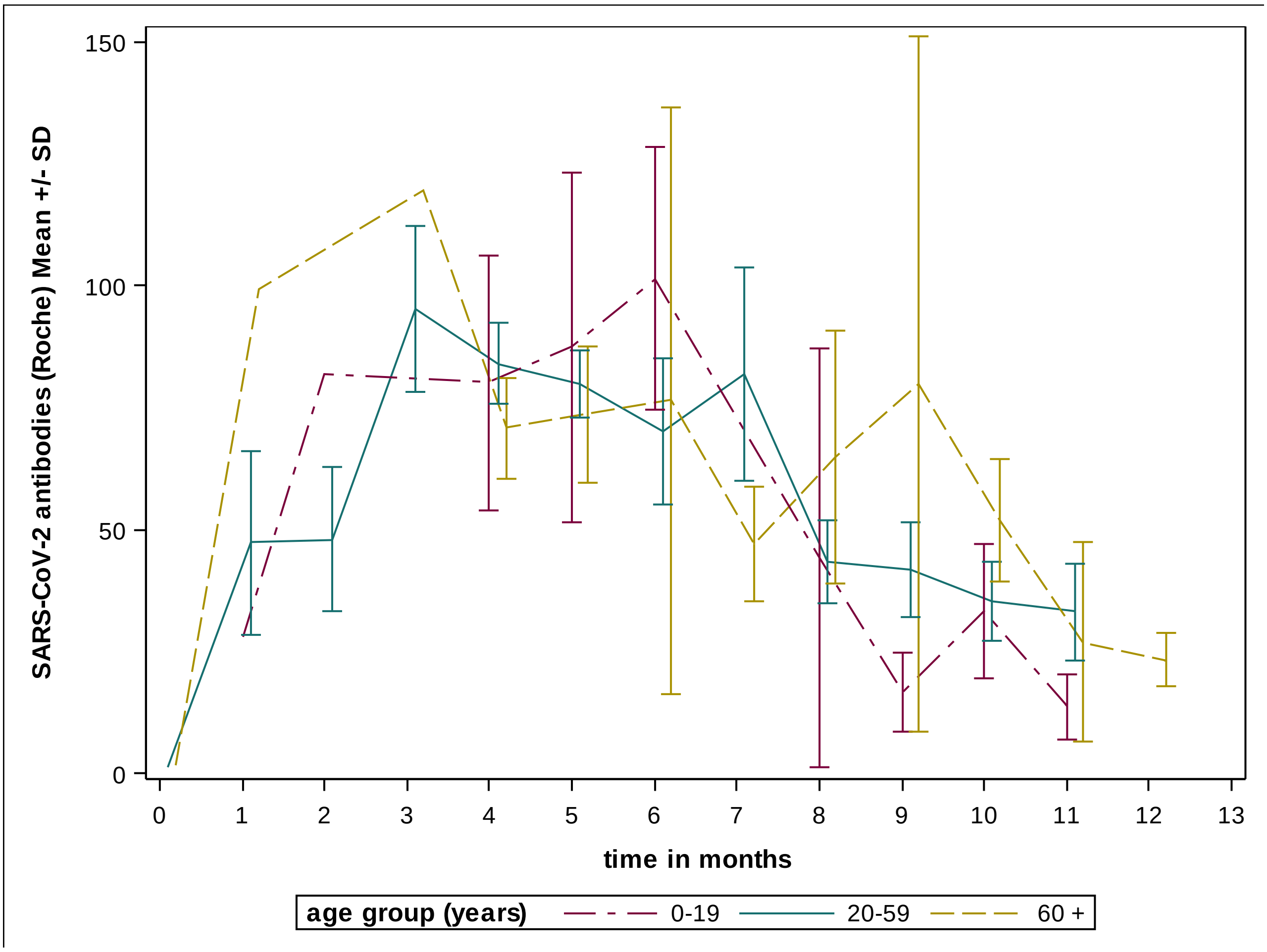

Suppl. Figure S1. Anti-SARS-CoV-2 Total Ig Roche – summary statistics by age category

Suppl. Table S1 shows the division into the age groups with the goal of differentiating between children and adolescents, adults and elderly populations. The division into three individual categories was as follows: Age 0-19, children + adolescents: 11.82%; Age 20-59, adults: 67.27%; Age 60+, elderly population: 20.91%.

Suppl. Table S1. Anti-SARS-CoV-2 Total Ig Roche – summary statistics divided into age category

Analysis Variable: SARS-CoV-2 antibodies (Roche)							
age group (years)	time in months	N	Mean	Std Dev	Median	Minimum	Maximum
0-19	1	1	27.9	.	27.9	27.9	27.9
	2	1	81.9	.	81.9	81.9	81.9
	4	4	80.1	52.4	79.1	18.9	143.4
	5	4	87.4	71.3	85.2	2.3	176.8
	6	6	101.4	66.1	98.4	35.2	189.8
	8	2	44.2	60.8	44.2	1.3	87.2
	9	3	16.6	13.9	14.4	3.9	31.4
	10	4	33.1	27.5	30.6	8.9	62.6
	11	4	13.7	13.0	11.8	0.6	30.5
20-59	0	1	1.5	.	1.5	1.5	1.5
	1	5	47.3	42.2	40.1	8.4	118.8
	2	7	48.0	39.1	37.6	8.8	104.6
	3	5	95.3	37.8	112.8	34.3	124.4
	4	24	84.1	39.8	80.5	2.9	146.9
	5	39	79.7	43.4	83.8	4.6	177.2
	6	10	70.1	47.6	63.4	9.9	169.4
	7	9	81.9	65.4	65.6	10.9	195.5
	8	14	43.4	32.5	39.6	1.6	108.9
	9	21	41.7	44.7	27.8	5.4	180.0
	10	18	35.3	34.3	23.5	2.2	121.7
	11	9	33.2	29.4	33.3	2.1	92.1
60 +	0	1	1.7	.	1.7	1.7	1.7
	1	1	99.2	.	99.2	99.2	99.2
	3	1	119.4	.	119.4	119.4	119.4
	4	8	70.9	29.4	67.4	21.7	121.7
	5	12	73.7	48.2	70.7	4.7	136.0
	6	2	76.5	85.0	76.5	16.4	136.6
	7	3	47.0	20.1	37.3	33.6	70.1
	8	7	65.0	68.7	23.1	4.3	186.6
	9	2	79.8	100.9	79.8	8.4	151.1
	10	10	51.9	39.4	47.0	10.5	130.1
	11	2	27.0	28.9	27.0	6.5	47.4
	12	2	23.3	7.8	23.3	17.8	28.8

Suppl. Figure S2 is a visualization of the mean course plot of antibody levels divided by gender. There are differences between the course of antibody response of males and females. These differences, however, were not statistically significant.

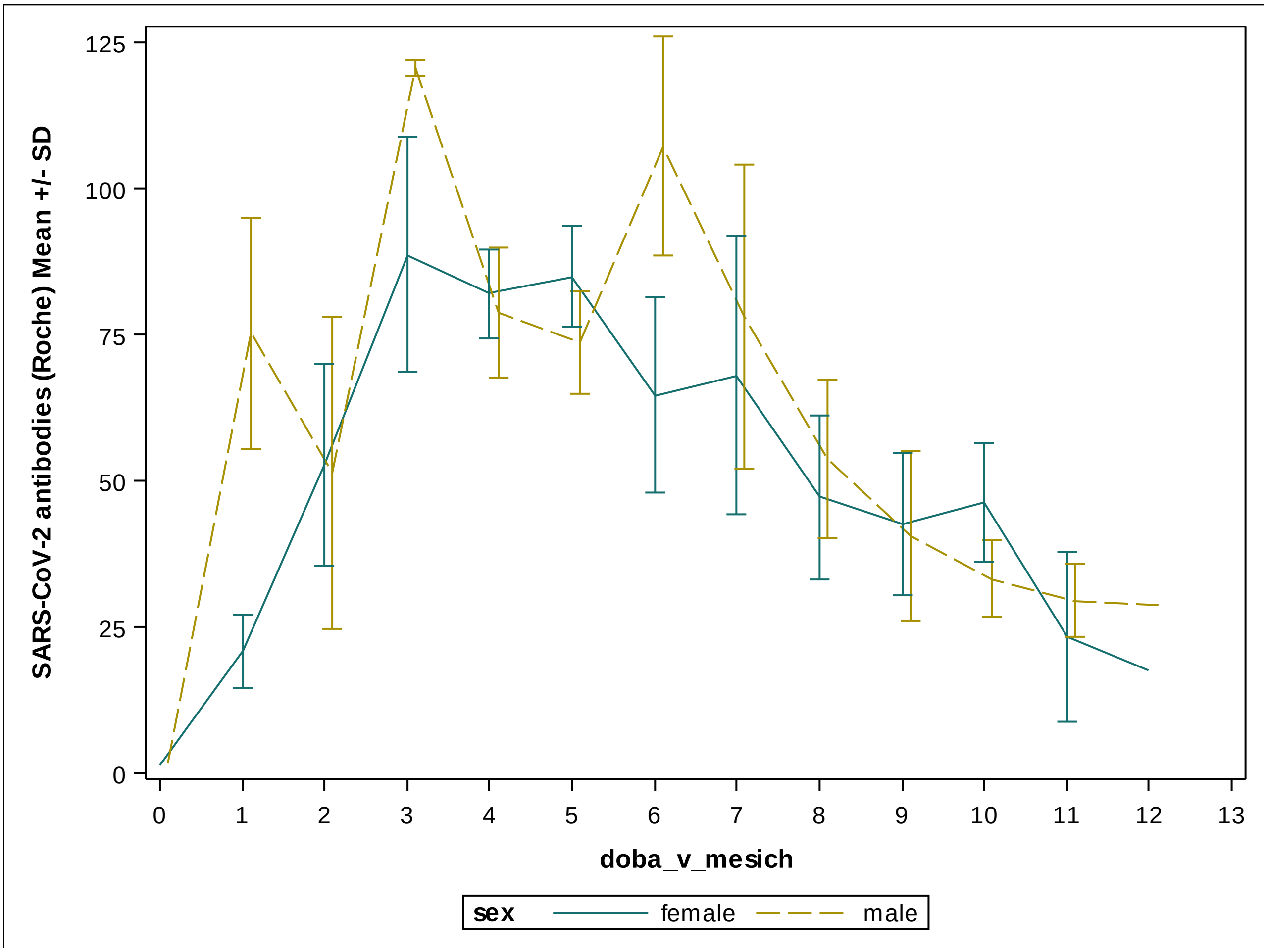

Suppl. Figure S2. Anti-SARS-CoV-2 Total Ig Roche – summary statistics by gender

Suppl. Table S2 shows the division by gender. The division into two categories was as follows: 53.6% males; 46.4% females.

Suppl. Table S2. Anti-SARS-CoV-2 Total Ig Roche – summary statistics by gender

Analysis Variable: SARS-CoV-2 antibodies (Roche)							
sex	time in months	N	Mean	Std Dev	Median	Minimum	Maximum
female	0	1	1.5	.	1.5	1.5	1.5
	1	3	20.9	10.8	26.4	8.4	27.9
	2	5	52.8	38.6	37.6	16.0	104.6
	3	4	88.7	40.2	98.0	34.3	124.4
	4	21	82.1	35.3	80.3	2.9	146.9
	5	26	84.9	44.0	92.3	2.3	149.7
	6	11	64.7	55.3	48.7	9.9	189.8
	7	6	68.1	58.5	49.6	24.9	179.0
	8	13	47.2	51.0	33.6	1.3	186.6
	9	15	42.6	47.5	31.4	8.4	180.0
	10	17	46.3	41.7	29.1	2.2	130.1
	11	6	23.5	35.5	7.7	0.6	92.1
	12	1	17.8	.	17.8	17.8	17.8
male	0	1	1.7	.	1.7	1.7	1.7
	1	4	75.3	39.8	71.1	40.1	118.8
	2	3	51.4	46.1	45.0	8.8	100.3
	3	2	120.7	1.8	120.7	119.4	121.9
	4	15	78.8	43.7	69.5	18.9	143.4
	5	29	73.7	47.4	73.5	4.6	177.2
	6	7	107.3	49.8	122.8	37.6	169.4
	7	6	78.2	63.9	66.9	10.9	195.5
	8	10	53.7	42.8	36.2	4.3	108.9
	9	11	40.6	48.5	13.0	3.9	151.1
	10	15	33.3	25.4	34.7	3.6	82.1
	11	9	29.6	19.0	33.3	6.5	63.7
	12	1	28.8	.	28.8	28.8	28.8

Suppl. Table S3 shows the individual data of all 110 patients involved in the study. The lines for each patient correspond with the number of samples taken from them. The age, sex and level of anti-SARS-CoV-2 Total Ig columns are followed by the number of days and months which passed from the positive PCR test. The last column shows the date of the positive PCR test.

Suppl. Table S3. Individual patient data (they are also provided in the extra excel file).

Patient No	Age	Sex	anti-SARS-CoV-2 Total Ig Roche	Time in days from positive PCR test	Time in month from positive PCR test	Positive PCR test
1	61	male	40,07	146	5	25.03.2020
1		male	22,27	232	8	
1		male	6,50	330	11	
2	47	female	48,73	188	6	18.03.2020
2		female	12,72	335	11	
3	50	male	78,31	188	6	18.03.2020
3		male	34,98	335	11	
4	63	male	65,29	121	4	14.04.2020
4		male	37,31	213	7	
5	53	female	80,28	121	4	14.04.2020
5		female	24,89	213	7	
6	50	male	18,89	138	5	10.04.2020
6		male	3,62	315	10	
7	49	female	44,51	138	5	10.04.2020
7		female	7,38	315	10	
8	51	female	108,30	139	5	28.03.2020
8		female	121,70	301	10	
9	65	male	92,27	145	5	01.04.2020
9		male	51,11	314	10	
10	61	female	120,10	145	5	01.04.2020
10		female	29,14	314	10	
11	49	male	40,17	150	5	15.03.2020
11		male	7,93	275	9	
12	39	female	107,30	150	5	15.03.2020
12		female	34,01	275	9	
13	54	male	89,16	167	5	07.03.2020
13		male	33,29	333	11	
14	42	female	1,48	0	0	30.09.2020
14		female	8,39	20	1	
14		female	23,89	58	2	
15	62	male	4,72	148	5	14.04.2020
15		male	4,26	230	8	
16	53	female	2,91	125	4	26.03.2020
16		female	1,61	244	8	
17	67	male	1,72	14	0	23.09.2020
17		male	99,17	37	1	
17		male	119,40	86	3	
18	20	female	80,71	136	4	10.03.2020
18		female	22,99	241	8	
19	58	male	68,32	138	5	14.03.2020
19		male	26,79	250	8	
20	59	female	73,29	138	5	14.03.2020
20		female	27,39	250	8	
21	73	male	52,07	126	4	23.04.2020
21		male	10,70	295	10	
22	66	female	64,19	126	4	23.04.2020
22		female	130,10	295	10	

23	45	female	96,63	126	4	23.04.2020
23		female	28,10	295	10	
24	57	male	107,90	164	5	21.03.2020
24		male	37,42	321	11	
25	54	female	139,10	164	5	21.03.2020
25		female	92,13	321	11	
26	47	male	118,80	45	1	11.08.2020
26		male	123,30	134	4	
26		male	122,80	190	6	
27	62	male	121,70	124	4	17.04.2020
27		male	42,93	293	10	
28	56	female	145,00	110	4	15.04.2020
28		female	72,89	233	8	
29	42	female	146,90	121	4	30.03.2020
29		female	93,16	240	8	
30	47	female	132,40	144	5	27.03.2020
30		female	100,20	307	10	
31	58	male	43,04	42	1	15.10.2020
31		male	33,78	127	4	
32	53	male	97,85	139	5	24.03.2020
32		male	45,59	240	8	
32		male	19,34	336	11	
33	14	female	94,75	118	4	08.04.2020
33		female	57,73	195	6	
33		female	31,41	286	9	
34	42	female	69,38	118	4	08.04.2020
34		female	42,18	195	6	
34		female	16,69	286	9	
35	19	female	63,52	118	4	08.04.2020
35		female	35,23	195	6	
35		female	14,36	286	9	
36	51	male	63,75	129	4	27.03.2020
36		male	13,00	262	9	
37	55	female	140,10	129	4	27.03.2020
37		female	116,80	262	9	
38	48	male	8,84	48	2	08.08.2020
38		male	4,59	167	5	
39	47	female	79,96	125	4	31.03.2020
39		female	33,56	247	8	
40	58	male	83,84	142	5	15.03.2020
40		male	45,31	268	9	
41	53	female	130,80	142	5	15.03.2020
41		female	58,83	268	9	
42	52	male	117,90	112	4	21.04.2020
42		male	177,20	157	5	
42		male	69,07	275	9	
43	25	male	31,79	111	4	29.04.2020
43		male	28,67	149	5	
43		male	10,70	267	9	
44	44	female	63,82	113	4	20.04.2020
44		female	60,13	158	5	
44		female	16,08	276	9	
45	44	female	106,10	136	4	21.03.2020
45		female	51,35	264	9	
46	47	male	112,80	145	5	29.03.2020
46		male	13,29	310	10	
47	15	male	18,90	112	4	01.05.2020

47		male	3,91	277	9	
48	59	male	100,00	126	4	27.03.2020
48		male	108,90	242	8	
49	38	female	48,02	114	4	28.04.2020
49		female	8,97	277	9	
50	43	female	89,91	151	5	16.03.2020
50		female	17,86	311	10	
51	70	male	77,96	123	4	29.03.2020
51		male	136,60	184	6	
51		male	151,10	263	9	
52	69	female	21,71	123	4	29.03.2020
52		female	16,43	184	6	
52		female	8,43	263	9	
53	47	male	40,05	43	1	24.09.2020
53		male	100,30	61	2	
54	49	female	42,97	147	5	18.03.2020
54		female	18,02	309	10	
55	11	female	189,80	184	6	12.04.2020
55		female	62,57	317	10	
56	41	female	83,01	184	6	12.04.2020
56		female	12,86	317	10	
57	52	male	19,20	120	4	20.04.2020
57		male	14,92	154	5	
57		male	5,40	285	9	
58	12	female	139,10	188	6	18.03.2020
58		female	30,52	344	11	
59	43	female	20,22	181	6	12.03.2020
59		female	2,11	350	11	
60	45	male	169,40	188	6	18.03.2020
60		male	63,66	344	11	
61	19	female	27,85	37	1	16.09.2020
61		female	81,91	64	2	
62	59	male	64,67	139	5	20.03.2020
62		male	103,90	262	9	
63	56	female	26,09	140	5	19.03.2020
63		female	9,10	263	9	
64	62	male	136,00	153	5	19.03.2020
64		male	73,90	313	10	
65	60	female	108,30	152	5	20.03.2020
65		female	69,13	312	10	
66	67	male	49,19	155	5	25.03.2020
66		male	11,72	316	10	
67	65	female	134,30	155	5	25.03.2020
67		female	89,68	316	10	
68	38	female	82,01	119	4	21.04.2020
68		female	49,58	153	5	
68		female	11,08	284	9	
69	42	female	34,30	103	3	30.04.2020
69		female	5,35	242	8	
70	64	male	21,96	139	5	12.03.2020
70		male	9,27	249	8	
71	56	female	99,45	139	5	12.03.2020
71		female	66,72	249	8	
72	49	female	75,30	119	4	24.04.2020
72		female	49,06	284	9	
73	62	male	70,12	220	7	02.03.2020
73		male	28,80	361	12	

74	61	female	33,57	220	7	02.03.2020
74		female	17,75	361	12	
75	49	female	143,80	142	5	09.03.2020
75		female	48,11	252	8	
76	15	male	81,56	152	5	12.04.2020
76		male	8,85	302	10	
77	40	male	85,25	153	5	11.04.2020
77		male	18,85	303	10	
78	10	male	176,80	153	5	11.04.2020
78		male	50,54	303	10	
79	46	female	78,80	128	4	15.04.2020
79		female	32,83	286	9	
80	62	female	41,12	151	5	10.04.2020
80		female	10,49	315	10	
81	23	male	30,46	137	5	20.03.2020
81		male	10,90	214	7	
81		male	3,67	305	10	
82	48	male	127,40	137	5	20.03.2020
82		male	91,53	214	7	
82		male	34,74	305	10	
83	49	female	114,50	137	5	20.03.2020
83		female	78,12	214	7	
83		female	31,43	305	10	
84	57	male	143,10	131	4	10.04.2020
84		male	48,26	291	10	
85	27	male	45,00	70	2	22.04.2020
85		male	195,50	204	7	
86	33	female	26,40	19	1	15.10.2020
86		female	104,60	46	2	
87	71	male	69,47	130	4	15.03.2020
87		male	101,00	228	8	
88	53	female	37,63	65	2	25.04.2020
88		female	27,71	200	7	
89	26	female	16,00	54	2	08.05.2020
89		female	9,89	188	6	
90	65	male	113,30	151	5	19.03.2020
90		male	108,20	242	8	
90		male	47,43	334	11	
91	45	female	83,14	81	3	10.04.2020
91		female	179,00	215	7	
92	37	male	73,45	141	5	26.03.2020
92		male	82,06	303	10	
93	11	female	88,77	137	5	20.04.2020
93		female	10,62	313	10	
94	47	female	72,15	137	5	20.04.2020
94		female	46,00	313	10	
95	47	male	103,50	163	5	30.03.2020
95		male	45,15	312	10	
96	17	female	2,29	148	5	30.03.2020
96		female	1,27	238	8	
96		female	0,57	325	11	
97	44	female	17,45	148	5	30.03.2020
97		female	7,83	238	8	
97		female	2,75	325	11	
98	30	female	112,80	98	3	10.04.2020
98		female	149,70	152	5	
99	65	male	23,11	143	5	07.03.2020

99		male	23,11	244	8	
100	53	male	57,41	170	6	09.03.2020
100		male	27,76	282	9	
101	15	male	37,57	170	6	08.03.2020
101		male	6,94	331	11	
102	4	male	149,00	170	6	08.03.2020
102		male	16,65	331	11	
103	43	female	16,74	151	5	21.03.2020
103		female	2,22	318	10	
104	51	male	67,98	143	5	15.03.2020
104		male	8,39	275	9	
105	48	female	94,71	141	5	17.03.2020
105		female	180,00	273	9	
106	17	male	143,40	136	4	17.03.2020
106		male	87,21	255	8	
107	48	male	121,90	105	3	17.04.2020
107		male	63,66	224	7	
108	45	female	124,40	105	3	17.04.2020
108		female	65,60	224	7	
109	43	female	88,92	133	4	18.03.2020
109		female	69,42	190	6	
109		female	46,52	252	8	
110	73	female	94,52	133	4	26.03.2020
110		female	186,60	258	8	
